# Vibration of Piezoelectric ZnO-SWCNT Nanowires

**DOI:** 10.3390/nano6120242

**Published:** 2016-12-15

**Authors:** Yao Xiao, Chengyuan Wang, Yuantian Feng

**Affiliations:** Zienkiewicz Centre for Computational Engineering, College of Engineering, Swansea University, Bay Campus, Fabian Way, Swansea, Wales SA2 8EN, UK; 599275@swansea.ac.uk (Y.X.); y.feng@swansea.ac.uk (Y.F.)

**Keywords:** carbon nanotubes, zinc oxide coating layer, hybrid nanowires, vibration, piezoelectric effect, inter-phase van der Waals interaction, Euler beam model

## Abstract

A hybrid nanowire (HNW) was constructed by coating a single-wall carbon nanotube (SWCNT) with piezoelectric zinc oxide (ZnO). The two components of the HNW interact with each other via the van der Waals (vdW) force. This paper aims to study the effect of the piezoelectricity in the ZnO layer and the inter-phase vdW interaction on the fundamental vibration of the HNWs. In doing this, a new model was developed where the two components of the HNWs were modeled as Euler beams coupled via the interphase vdW interaction. Based on the model, the dependence of the frequency on an applied electrical voltage was calculated for HNWs of different geometric sizes to reveal the voltage effect. The results were then compared with those calculated without considering the inter-phase vdW interaction. It was found that the interphase vdW interaction can substantially decrease the structural stiffness, leading to a greatly enhanced piezoelectric effect but a lower frequency for the vibration of the HNWs.

## 1. Introduction

**Figure 1:**
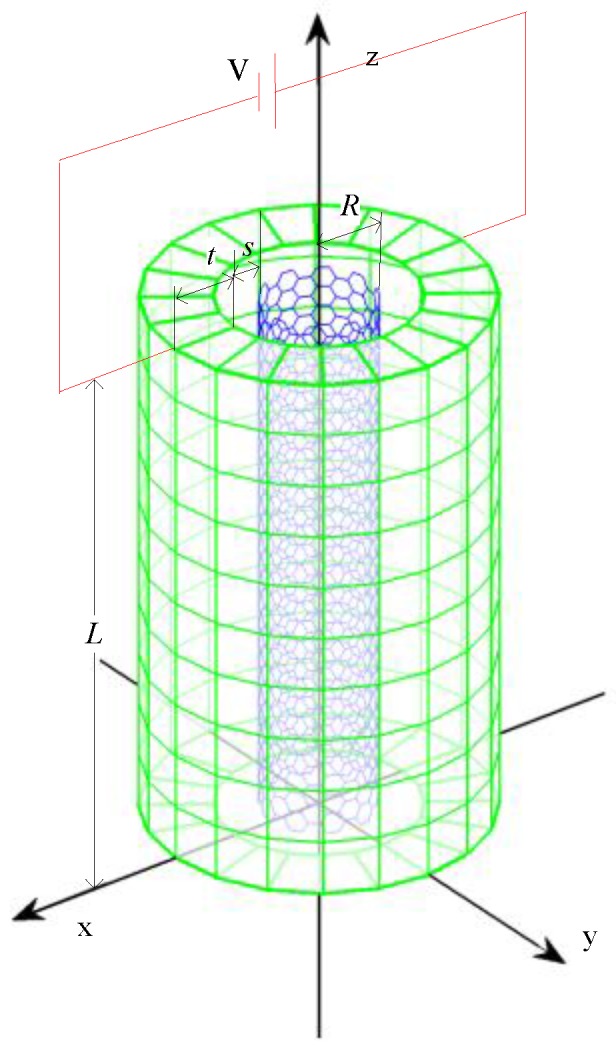
Schematic illustration of the hybrid nanowire (HNW) structure where a single-wall carbon nanotube (SWCNT) of radius *R* is coated by a cylindrical layer of ZnO of thickness *t*. The SWCNT and ZnO layer are bonded via the van der Waals (vdW) interaction with equilibrium interspacing *s*.

A hybrid nanowire (HNW) (Figure 1) was fabricated by coating carbon nanotubes (CNTs) with a layer of zinc oxide (ZnO) crystalline [1,2,3,4,5]. This novel nanomaterial is promising for the elements of nanosensors/actuators, nanoresonators, nanogenerators and smart nanocomposites due to the synergy of stiff and electrically conducting CNTs, and the piezoelectric and semiconducting ZnO layer. The performance of the HNW-based nanodevices/materials depends critically on the electromechanical responses of HNWs. Thus, the coupling between the mechanics of HNWs and the piezoelectricity of the ZnO layer has attracted increasing attention in recent research. Indeed, the piezoelectric effect on nanomaterials has been one of the fundamental issues in the area of nanomechanics [6,7,8,9,10,11,12]. Here it should be pointed out that different from conventional composite materials, nanocomposites such as HNWs consist of nanoscale fillers and a matrix which are normally bonded with each other via the van der Waals (vdW) interaction without chemical bonds. This unique feature of nanocomposites has excited a series of studies in the last decade [13,14,15,16]. In 2006, Jiang et al. first studied the behavior of the interface between CNTs and the matrix based on the Lennard-Jones (L-J) potential [13]. Using the same model, Tan et al. further investigated the effect of the interface on the overall mechanical responses, especially the CNT-matrix interface debonding of the nanocomposites [14]. In 2013, Zhao and his co-workers studied the interface between graphene/CNT and the substrate by using the L-J potential [15]. Very recently, the same group paid attention to the functionally graded interphase (FGI) between CNTs and the matrix [16] or spherical fillers and coating materials. Efforts were made to examine the effect of the FGI layer on the overall mechanical behavior of the hybrid system [17]. With the assumption of a diluted solution, a 3D solution was obtained for the constitutive relation of the nanofiller-matrix/coating layer interface when the nanocomposites were subjected to a hydrostatic tension. The strength was also evaluated for the interface of the composites. Nevertheless, in previous studies of the HNWs, the interphase vdW interaction was neglected completely. As a result, its effect on the overall electromechanical responses has never been investigated for HNWs. In addition, as pointed out in [18], the small-scale effect will lead to stiffening of piezoelectric NWs, which, however, may reduce their piezoelectric effect as far as very thin NWs are concerned. This is in contrast to the classical theory (without the small-scale effect) which predicts a higher piezoelectric effect for smaller NWs. It is thus of great interest to re-examine the small-scale effect on the performance of HNWs. This situation indeed provides an impetus to study the electromechanical behavior of HNWs by considering the interphase vdW interaction as a small-scale effect.

In the present work, attention was focused on the fundamental vibration of the HWNs by considering the piezoelectric effect of the ZnO layer and the interface between the CNTs and ZnO layer. The emphases were placed on the effect of the externally applied voltage and the interphase vdW interaction on the vibrational responses of HWNs. The paper is organized in the following way. In Section 2 the electromechanical model is introduced to account for the piezoelectric effect of the ZnO layer and the CNT-ZnO interphase vdW interaction. The dynamics equations are then derived for the HWNs based on the simple Euler beam theory. Section 3 shows the results given by the models established in Section 2, where the effect of the voltage and the vdW interaction are of major concern. The conclusions are finally drawn in Section 4.

## 2. Methodology

As shown in Figure 1, a HNW is comprised of an inner single CNT (SWCNT) of radius *R* and a ZnO coating layer of thickness *t*. The length *L* of the HNW is assumed to be the same as those of the SWCNT and ZnO layer (Figure 1). The SWCNT interacts with the ZnO layer with the interphase vdW interaction which is characterized by the Lennard-Jones (L-J) 6–12 potential.

(1) $$V\left(r\right)=4\varepsilon \left({\left(\frac{\sigma }{r}\right)}^{12}-{\left(\frac{\sigma }{r}\right)}^{6}\right)$$

where $\sqrt[{6}]{2}\sigma$ is the equilibrium distance between the atoms, ε is the depth of potential and *r* is the distance between two reference points. For the ZnO-CNT interaction the two constants can be estimated by $\sigma_{\mathrm{ZnO}-\mathrm{CNT}}=\frac{1}{2}\left(\sigma_{\mathrm{ZnO}-\mathrm{ZnO}}+\sigma_{C-C}\right)$ and $\varepsilon_{\mathrm{ZnO}-\mathrm{CNT}}=\sqrt{\varepsilon_{\mathrm{ZnO}-\mathrm{ZnO}}{\text{×}\varepsilon }_{C-C}}$ where the constants of the L-J potential between C atoms are $\sigma_{C-C}=4A$ and $\varepsilon_{C-C}=0.0065\mathrm{eV}$ [19]. The constants of ZnO are not available but σ and ε of Au–O, Mg–O, Al–O, Ca–O, Fe–O, Si–O, K–O, Na–O are found to fall in the range of (3.67A, 2.267A) and (0.0024eV, 0.0180eV) [19]. It is thus reasonable to assume that $\sigma_{\mathrm{ZnO}-\mathrm{ZnO}}$ and $\varepsilon_{\mathrm{ZnO}-\mathrm{ZnO}}$ are of the order of magnitude 3A and 0.01eV, respectively. Then based on the above mentioned equations we obtained $\sigma_{\mathrm{ZnO}-C}\approx 3.5A$ and $\varepsilon_{\mathrm{ZnO}-C}\approx 0.008\mathrm{eV}$. Following a similar procedure in [13] the cohesive potential at the CNT-ZnO interface due to the vdW interaction can be derived as:

(2) $$\Phi =2\pi \rho_{cnt}\rho_{zno}R\int_{0}^{L}\left(\int_{R+s}^{R+s+t}\int_{0}^{2\pi }\int_{0}^{L}V\left(r\right)\lambda d\theta \cdot dz_{zno}\cdot d\lambda \right)\cdot dz_{cnt}$$

where $\rho_{cnt}$ ($\left(2{.27g/\mathrm{cm}}^{3}\right)\times 0.34\mathrm{nm}$ [20,21]) and $\rho_{zno}$ (5.61g/cm^3^ [22]) are the mass densities of SWCNT (per unit area of lateral surface) and the ZnO layer (per unit volume), respectively; λ is the radius coordinator, z the axial coordinator and θ the angular coordinator of the cylindrical polar system. Here the subscripts ‘cnt ‘and ‘zno’ represent the parameters of SWCNT and the ZnO layer. In addition, $r=\sqrt{\gamma^{2}+R^{2}-2R\gamma \cos\theta_{zno}+{\left(z_{cnt}-z_{zno}\right)}^{2}}$ represents the distance between the differential element on the CNT and the one on the ZnO coating layer. The interphase vdW pressure *p* can then be calculated by:

(3) $$p={\frac{1}{2\pi RL}\cdot \frac{\partial \Phi }{\partial s}|}_{s=s_{eq}}$$

where $s_{eq}$ denotes the equilibrium inter-phase spacing associated with the lowest value of Φ (and zero vdW interaction), and 2πRL is approximately equal to the area of the SWCNT-ZnO interface provided that the HNW is long and thin, i.e., L>20R. For a very small deviation from the equilibrium interphase spacing $s_{eq}$ the vdW pressure $p\approx c\left(s-s_{eq}\right)=c\left(w_{cnt}-w_{zno}\right)$ where $w_{cnt}$ and $w_{zno}$ represent the transverse deflection of the inner SWCNT and the outer ZnO layer, respectively. The vdW interaction coefficient $c=1.136\times 10^{11}$ N/m and $c=0.943\times 10^{11}$ N/m can be evaluated based on Equations (2) and (3) for the SWCNTs of radius 0.68 nm and 2.51 nm, respectively. As will be shown below the coating thickness *t* > 10 nm is considered. In this case the influence of thickness *t* on *c* is small and thus was not considered in the present work.

In this work we considered long and thin HNWs where the constituent SWCNT and coating layer can be treated as two Euler beams whose dynamic equations are coupled via the vdW interaction terms. In addition, as shown in Figure 1 an electrical voltage *U* was applied in the transverse direction to the HWN.

(4) $$\left\{ \begin{array}{l} {\left(EI\right)}_{cnt}\frac{\partial^{4}w_{cnt}}{\partial x^{4}}+{\left(\rho S\right)}_{cnt}\frac{\partial^{2}w_{cnt}}{\partial \tau^{2}}=c\left(w_{zno}-w_{cnt}\right)\\ {\left(EI\right)}_{zno}\frac{\partial^{4}w_{zno}}{\partial x^{4}}+{\left(\rho S\right)}_{zno}\frac{\partial^{2}w_{zno}}{\partial \tau^{2}}=P_{eff}\frac{\partial^{2}w_{zno}}{\partial x^{2}}-c\left(w_{zno}-w_{cnt}\right) \end{array}\right.$$

here the equivalent bending stiffness of the SWCNT is ${\left(EI\right)}_{cnt}=\pi \cdot E_{cnt}\left(R^{2}+{\left(\frac{t_{cnt}}{2}\right)}^{2}\right)R\cdot t_{cnt}$ where $E_{cnt}=3.5\mathrm{TPa}$ is the equivalent Young’s modulus and $t_{cnt}=0.1\mathrm{nm}$ is the effective thickness of SWCNTs [23,24]. The bending stiffness of the ZnO beam is ${\left(EI\right)}_{zno}=\frac{\pi d^{4}}{64}\left(1-{\left(1-\frac{2t}{d}\right)}^{4}\right)\left(c_{11}+\frac{e_{31}^{2}}{k_{33}}\right)$ [8], where *d* =2(R+s+t) is the diameter of the HNW; c_11_ (207 GPa) is the elastic modulus, e_31_ (−0.51 $C\cdot m^{-2}$) is the piezoelectric constant and k_33_ (7.88 $\times 10^{-11}F\cdot m^{-1}$) is the dielectric constant of ZnO [25]. In addition, the effective axial force acting on the ZnO coating layer is [8]:

(5) $$P_{eff}=\pi \left(d-t\right)\cdot t\cdot \left(c_{11}\varepsilon_{0}+e_{31}\frac{U}{2t}\right)$$

where $\sigma_{x}^{0}$ is the residual axial stress in the ZnO layer, which is assumed to be zero in the present study. In Equation (4), τ is time, ${\left(\rho S\right)}_{cnt}=2\pi Rt_{cnt}\rho_{cnt}$ and ${\left(\rho S\right)}_{zno}=\pi \left(d-t\right)t\cdot \rho_{zno}$ are the mass densities per unit length of the SWCNT and the ZnO coating layer respectively.

For simply supported HNWs the solution to Equation (4) takes the form:

(6) $$w_{i}=A_{i}\cdot \sin\left(\frac{m\pi }{L}x\right)\cdot e^{i\omega t}\;(i=1,\;2)$$

where *A_1_* and *A_2_* are the vibration amplitudes of the SWCNT and the ZnO layer, respectively, *m* is the number of half wave number (or mode number) of the vibration, *x* is the axial coordinator, ω is the angular frequency and *i* is the imaginary unit. Substituting solution (6) into Equation (4) leads to a system of algebraic equations:

(7) $$M{\left(\omega ,c,m\right)}_{2\times 2}\left\lceil \begin{array}{l} A_{1}\\ A_{2} \end{array}\right\rceil =0$$

**T**he condition for nonzero solutions of *A_1_* and *A_2_* reads detM=0. Solving this characteristic equation yields the frequency f=ω/2π which is a function of the mode number *m*, the applied voltage *U* and the vdW interaction coefficient *c*.

## 3. Results and Discussions

In the present study, the technique demonstrated above was employed to calculate the vibration frequency of HNWs subjected to an electric voltage. The emphasis was placed on the influence of applied voltage *U* and the vdW interaction between the SWCNT and the ZnO layer. The details of the results and the discussions based on the results are shown in the following subsections.

### 3.1. Influence of the Piezoelectric Effect

**Figure 2:**
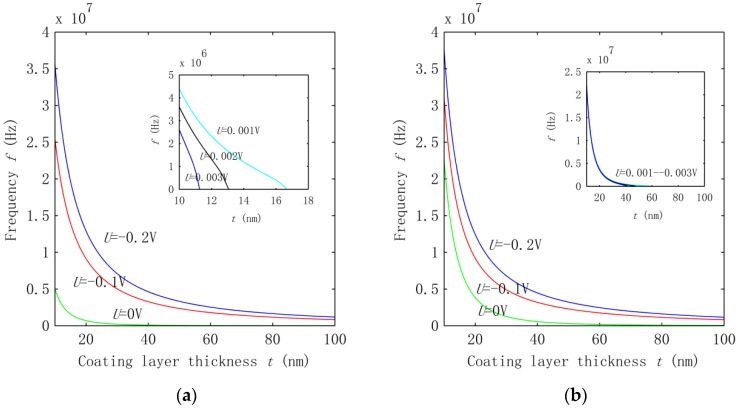
Frequencies of piezoelectric HNWs with interphase vdW interaction where the voltage applied is in the range of (−0.2 V, 0.003 V), and the inner SWCNT has a radius (**a**) *R* = 0.68 nm and (**b**) *R* = 2.51 nm. The insets show the results for *U* = 0.001, 0.002 and 0.003 V.

In this section, the effect of the applied voltage was examined for the vibration of the HNWs. To this end, we calculated the frequency of the fundamental mode for the HNWs where the coating thickness increases from 10 to 100 nm and the applied voltage varying in the range of (−2 V, 0.003 V). The results are shown in Figure 2a,b for SWCNT radii *R* = 0.68 nm and 2.51 nm, respectively. The aspect ratio of HWNs was fixed at *L*/(*R* + *t*) = 40, i.e., the length of the HNW increases with the rising radiu*s R* of the SWCNT and the thickness of the coating layer.

Figure 2a for the HNWs with a thin SWCNT indicates that, at a given voltage, the vibration frequency decreases with the growing coating thickness. The rate of the change, however, decreases monotonically with the increasing thickness *t* and becomes very small at *t* > 80 nm. This behavior can be understood as a result of the competition between the opposing effects of the rising second moment of the area (thickness increase) and the growing length of the HNW. Also, as seen from the figure, applying a negative external voltage −0.1 V and −0.2 V can substantially raise the frequency, showing the significant piezoelectric effect on the vibration of HNWs. For example, at *t* = 10 nm the frequency (*U* = 0) is raised by a factor of four or six when a voltage *U* of −0.1 V or −0.2 V is applied. When a positive voltage is considered, an opposing piezoelectric effect is observed in the inset of Figure 2a, where a greater voltage leads to a lower frequency. In particular, for a given voltage the frequency turns out to be zero when the coating thickness *t* increases up to a critical value. Such a critical value of *t* decreases from 16.6 nm to 13.1 nm and 11.3 nm when *U* is raised from 0.001 V, to 0.002 V and 0.003 V. A similar behavior is also observed in Figure 2b for the HNW with a large SWCNT. However, the effect of the voltage in Figure 2b becomes small as compared with the effect observed in Figure 2a. It is understood that, in general, the frequency change associated with the applied voltage is a result of the piezoelectricity-induced effective axial stress $e_{31}\frac{U}{2t}$. Here $e_{31}$ is negative. Thus, a negative voltage results in a tensile stress (positive) and increases the frequency, whereas a positive voltage leads to a compressive stress (negative) which decreases the structural stiffness and the frequency of the HNWs accordingly. When a positive voltage approaches a critical value, the induced compression finally exceeds the critical buckling load and leads to the instability of the HNW structures. In particular, the vibration frequency turns out to be zero at the onset of HNW buckling.

**Figure 3:**
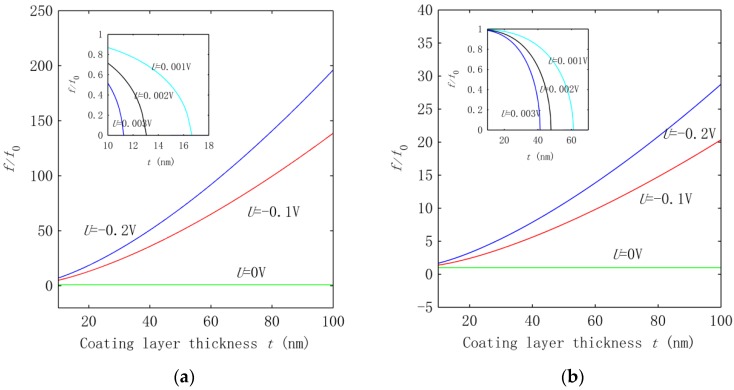
Frequency ratio $f/f_{0}$ calculated at *U* = −0.2, −0.1, 0 V. The inset shows the corresponding results associated with *U* = 0.001, 0.002, 0.003 V.

In addition to the tendency of the frequency change, we were also interested in measuring the above voltage effect on the frequency for the two groups of HNWs. Thus, a more detailed study was carried out to quantify the voltage effect based on the frequency ratio $f_{v}/f_{0}$, where $f_{v}$ is the frequency associated with voltage *U* and $f_{0}$ is the one calculated without applying a voltage. The results are summarized in Figure 3a,b for the HNWs studied in Figure 2a,b, respectively. Figure 3 shows that the effect of the voltage measured by the frequency ratio increases rapidly with the growing thickness of the ZnO coating layer. Consistent with the observation in Figure 2a, at *t* = 10 nm the frequency increases four or six times at *U* = −0.1 V or −0.2 V as far as the HNWs with a thin SWCNT were concerned. Increasing *t* to 100 nm leads to a frequency around two orders of magnitude higher than the value at *U* = 0. This trend remains qualitatively similar in Figure 3b for the group of HNWs with a large SWCNT, but the voltage effect becomes significantly smaller. For example, at *t* = 100 nm, the frequency $f_{0}$ up-shifts by only one order of magnitude in Figure 3b. From here it follows that for the same voltage and coating thickness, the voltage effect for the HNWs with the thin SWCNT is roughly an order of magnitude stronger than the effect on the HNWs with the large SWCNT.

In Figure 3, the voltage effect enhanced by increasing the thickness can be attributed to the fact that when the coating thickness grows, more piezoelectric material is involved in the HNWs. On the other hand, the smaller piezoelectric effect achieved for the HNWs with a large SWCNT is due to the improved transverse rigidity of the HNWs. Thus, for the piezoelectric HNWs, thinner SWCNTs and a thicker coating layer would greatly improve the sensitivity of the frequency to the applied voltage.

### 3.2. Effect of Interphase VdW Interaction 

**Figure 4:**
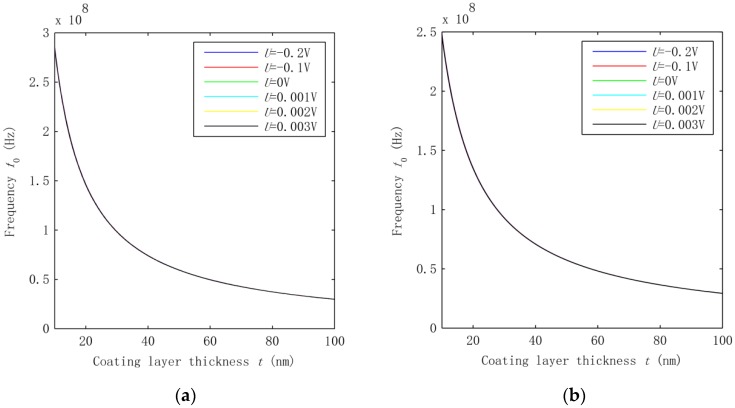
Frequencies calculated without considering the interphase vdW interaction for the HNWs where the SWCNT radius *R* is (**a**) 0.68 nm and (**b**) 2.51 nm, respectively.

As shown in Figure 1, HNWs have a hybrid structure which consists of the inner SWCNT and the outer coating layer. The two components are coupled with each other with the interphase vdW interaction. It is thus of geat interest to examine its effect on the fundemental vibration of the HNWs. To this end, we compared the frequency shown in Figure 2 where the vdW interaction is considered with those obtained without considering the vdW force. Thus, the frequency of the HNWs was calculated based on the mechanical model [22] where the interphase vdW interaction was neglected and the HNWs were considered as composite beams where a contiuum interface was assumed between the coating layer and the SWCNT. Similar to Figure 2, a voltage *U* is applied, ranging from −0.2 V to 0.003 V. The results are shown in Figure 4a,b for the HNWs where the SWCNT radius is 0.68 nm and 2.51 nm, respectively.

Comparisons between Figure 2 and Figure 4 show that the frequency in Figure 4 is up-shfited substantially while the thickness-dependence of the frequency remians qualitatively similar. On the other hand, the voltage effect on the HNWs becomes very small in Figure 4, which is in sharp contrast to the strong effect observed for the same HNWs in Figure 2 where the vdW interaction was taken into consideration. This comparison indeed revealed the effect of the vdW interaction on the vibration of the HNWs. The lower frequency in Figure 2 suggests that the vdW interaction leads to a lower structural stiffness of the HNWs. Thus, neglecting its effect may largely overestimate the frequency but underestimate the effect of the voltage-induced axial stress on the vibration of the HNWs. Indeed, the introduction of the vdW interaction allows the two components of HNWs to move relative to each other as two individual beams which would othewise vibrate as one single beam with the same deflection and a higher structural stiffness. At the same time, the effect of the voltage-induced axial stress turns out to be more substantial due to the lower structural stiffness in the presence of the interphase vdW interaction. Thus, the interphase vdW interaction in the HNWs offers a new avenue to largely improve the piezoelectric effect on the vibration of piezoelectric NWs. Here it is emphasized that the interphase vdW interaction is a unique feature of nanoscale structures, which, as shown before, would decrease the structural stiffness of HNWs and thus enhance their piezoelectric effect. In other words, for the hybrid structures considered in this study, the miniturization of structures to the nanoscale may lead to an improved pizeoelectric effect due to the decrease of the structural stiffness in the presence of the interphase vdW interaction.

**Figure 5:**
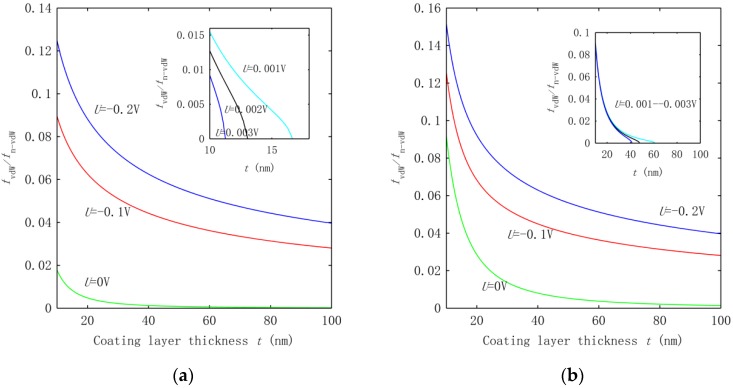
Frequency ratio $f_{vdw}/f_{n-vdw}$ calculated for the HWNs with the SWCNT radius *R* equal to (**a**) 0.68 nm and (**b**) 2.51 nm.

To quantatively measure the effect of the interphase vdW interaction, we calculated the freqeuncy ratio $f_{vdw}/f_{n-vdw}$ in Figure 5 where $f_{vdw}$ is the frequency obtained in the presence of the vdW interaction and $f_{n-vdw}$ is the one caculated in the absence of the vdW interaction. Figure 5 demonstrates that the existence of the interphase vdW interaction can down-shift the frequency by one to two orders of magnitude for the thickness *t* considered in the present study. In addition, the frequency ratio decreases with the increasing thickness in Figure 5, showing that a greater effect of the vdW interaction can be achieved for the HNWs with a larger coating thickness. Another phenomenon observed is that once the vdW interaction was introduced, the frequency became sensitive to the electrical voltage applied. This is in accordance with the analysis shown above. Since the frequency is directly related to the structural stiffness, the data in Figure 5 also measure the effect of the vdW interaction on the overall equivalent structural stiffness of the HNWs.

## 4. Conclusions 

In this study, the inner SWCNT and the outer ZnO coating layer of the piezoelectric HNWs were modeled as two Euler beams connected to each other via the vdW interaction. The vibration of the HNWs subjected to an applied electrical voltage was studied based on the above model to examine the effect of the piezoelectric coating layer and the interphase vdW interaction. The new findings are summarized as follows.

The vdW interaction can substantially reduce the structural stiffness of the hybrid structures and thus greatly down-shift the vibration frequency of the HNWs. This effect turns out to be stronger for the HNWs with a greater thickness of the coating layer but a fixed length-to-diameter aspect ratio. The effect, however, decreases when a SWCNT with a lager radius is used in the HNWs. Additionally, the stiffness reduction due to the vdW interaction can greatly enhance the effect of the axial stress generated by an electrical voltage via the piezoelectric effect of the ZnO layer. As a result, the piezoelectric effect or the effect of the voltage on the vibration can be improved substantially due to the introduction of the vdW interaction. In other words, compared with the homogenous piezoelectric nanowires, the vibration frequency of the HNWs changes much more sensitively with the electrical voltage applied. In particular, the vdW interaction may lead to early structural instability of HNWs at a small positive electrical voltage.
